# Novel avenues to control blood pressure: targeting the renal lymphatic system

**DOI:** 10.1042/CS20220775

**Published:** 2023-04-19

**Authors:** Andreia Zago Chignalia

**Affiliations:** 1 Department of Anesthesiology, The University of Arizona, College of Medicine – Tucson, AZ, U.S.A.; 2 Department of Physiology, The University of Arizona, College of Medicine - Tucson, AZ, U.S.A.; 3 Department of Pharmacology and Toxicology, The University of Arizona, College of Pharmacy – Tucson, AZ, U.S.A.; 4 Sarver Heart Center, The University of Arizona, College of Medicine - Tucson, AZ, U.S.A.

**Keywords:** endothelial cells, hypertension, lymphangiogenesis, renal physiology

## Abstract

Hypertension is associated with the activation of the immune and lymphatic systems as well as lymphangiogenesis. The changes in the lymphatic system are considered an adaptive response to mitigate the deleterious effects of immune and inflammatory cells on the cardiovascular system. In the article recently published in *Clinical Science* by Goodlett and collaborators, evidence is shown that inducing renal lymphangiogenesis after the establishment of hypertension in mice is an effective maneuver to reduce systemic arterial blood pressure. In this commentary, we will briefly review what is known about the relationship between the activation of the immune and lymphatic systems, and the resulting effects on systemic blood pressure, summarize the findings published by Goodlett and collaborators, and discuss the impact of their findings on the field.

Hypertension is a multifactorial syndrome that affects about 46% of the adult population in the United States [1]. In 95% of the cases, hypertension is presented in its essential form, i.e., with unknown origin [2]. The processes underlying the development of essential hypertension include the increased production of reactive oxygen species, higher sympathetic tone, pronounced activation of the renin-angiotensin-aldosterone system, the release of inflammatory factors, and the activation of the immune system. All these processes are part of an adaptive response that can result in the development of cardiovascular diseases and end-organ damage.

Although hypertension has been extensively studied and several pharmacological treatments are available to control blood pressure levels, the incidence of hypertension continues to rise [3]. This suggests that the broad range of processes underlying the pathology of hypertension cannot always be balanced, compromising the control of blood pressure levels. Thus, advancing the understanding of the relative contribution of isolated processes underlying hypertension is needed as it may represent the next step on treating high blood pressure.

It is well established that the activation of the immune system is part of the pathogenesis of hypertension. Founding studies described that the injection of normal or infarcted renal tissue in normotensive rats resulted in the increase in blood pressure, evidencing a role for immune response to the control of blood pressure levels [4]. A series of studies showed that the treatment of hypertensive animals with mycophenolate mofetil (MMF), an immunosuppressive drug, can reduce blood pressure in rodents. MMF was shown to reduce blood pressure levels in Dahl salt-sensitive rats [5] and to prevent the development of salt-induced hypertension in ‘two-hit’ models of hypertension in mice. Specifically, MMF did not prevent the development of hypertension during chronic infusion of angiotensin [6] or L-NAME treatment [7] but prevented the development of hypertension when these mice were further challenged with a high salt diet. A positive effect of MMF in the reduction in blood pressure was also observed in spontaneous hypertensive rats in a salt-independent manner [8]. Importantly, MMF also reduced blood pressure in a small group of hypertensive patients [9], showing the importance of the immune system for the regulation of blood pressure in humans. It was later shown that T lymphocytes are specifically required to the development of hypertension in Angiotensin-II and DOCA-salt models of hypertension [10], shedding light on the relevance of specific cell populations as a regulator of systemic blood pressure.

The contribution of the immune system to hypertension is complex and it involves the activation of many of the immune cell types, including lymphocytes T and B, dendritic cells, and leukocytes [11]. As a result of such widespread activation, the lymphatic system, known to transport immune cells to organs in need is ‘recruited’ not only to assist with the delivery of immune and inflammatory cells to target tissues but also to help clear the accumulation of such cell types to prevent localized damage to the vasculature.

The activation of the lymphatic system is so pronounced during hypertension that it is manifested in two ways: through the expansion of the lymphatic system via lymphangiogenesis and via the presentation of antigens by endothelial lymphatic cells, a mechanism that removes immune cells from the circulation to the draining lymph nodes as a clearance system. An association between high blood pressure and lymphangiogenesis has been previously established in models of hypertension in which renal function is compromised [12–14] as well as during sodium homeostasis [15]. This is particularly important as approximately 50% of hypertensive patients have salt-sensitive hypertension. Moreover, patients with salt-sensitive hypertension are at increased risk to develop chronic kidney disease and to suffer cardiac events [13]. Whether lymphangiogenesis occurs during hypertensive states in which sodium handling is not affected is unclear and should be investigated.

Renal lymphangiogenesis has been suggested as a pathway to decrease blood pressure levels in mice [16]. This seems to be an organ-specific phenomenon as skin lymphangiogenesis does not alter blood pressure during salt-induced hypertension [17]. In the present study, mice-deficient in dermal lymphatics (K14-VEGFR3), or with increased dermal lymphatic vessels (K14-VEGF-C) were exposed to high-salt diet or DOCA+ L-NAME + high-salt diet models of hypertension. It was observed that high-salt diet only was insufficient to induce increases in blood pressure in both studied mouse strains. In contrast, after exposure to the DOCA-salt model, both strains developed hypertension and sodium deposition in skin and muscle in a similar way than wild-type mice. Thus, altering the dermal lymphatic system does not affect the systemic regulation of blood pressure.

The findings of Goodlett and colleagues [18] recently published in *Clinical Science* advance the field by showing evidence that the genetic induction of renal lymphangiogenesis after the establishment of hypertension is an efficient approach to restore normal blood pressure levels in mice. The study elegantly used three different models of hypertension induced either by angiotensin II (A2HTN), the inhibition of endothelial nitric oxide synthase (eNOS) (LHTN) or the inhibition of eNOS followed by a washout period and the addition of 4% salt to the diet (SSHTN). Hypertension was induced in mice with inducible kidney-specific overexpression of VEGF-D (KidVD+) and respective controls KidVD-. After one week of specific treatment, hypertension was established and renal lymphangiogenesis was induced using doxycycline. Next, blood pressure, immune and inflammatory cells infiltration as well as sodium handling were monitored for four weeks.

The work published by Goodlett and collaborators in *Clinical Science* [18] complements previous work from the same group where they have observed that when renal lymphangiogenesis is genetically induced, KidVD+ mice fail to develop hypertension when challenged with A2HTN [14], LHTN [13], and SSHTN [13] models of hypertension. In the present study, they demonstrate that the reduction in blood pressure in all three models of hypertension seem to share a basic mechanism adaptation to increase sodium clearance as shown by an increase in the fractional excretion of sodium (FENa) that is associated with the decrease expression of the sodium transporters NCC (sodium-chloride symporter), Nhe3 (sodium-hydrogen exchanger 3), and ENaC (epithelium sodium channel) in the A2HTN and SSHTN models of hypertension but not in the LHTN model of hypertension. This was not associated with additional changes in renal function as assessed by the levels of creatinine, potassium, and chloride in the urine.

The changes in sodium handling are associated with changes in specific populations of immune cells. The common changes in cells populations observed in the three tested models of hypertension were: an increase in CD45+ cells (global marker for leukocytes); a decrease in activated dendritic cells (F4/80+/CD11c+/CD38+) and CD11b+ myeloid cells. Additionally, the expression of TGFβ1 and TGFβ3 was increased across the three models of hypertension. On contrary, some cell populations were changed only in the models where the expression of sodium transporters was altered and they include an increase in CD4+ helper T cells, CD8+ cytotoxic T cells, and CD11b+/F480+CD206CD11c+ macrophages and a decrease in CD4+CD62L+CD44+ memory T cells (Figure 1).

**Figure 1:**
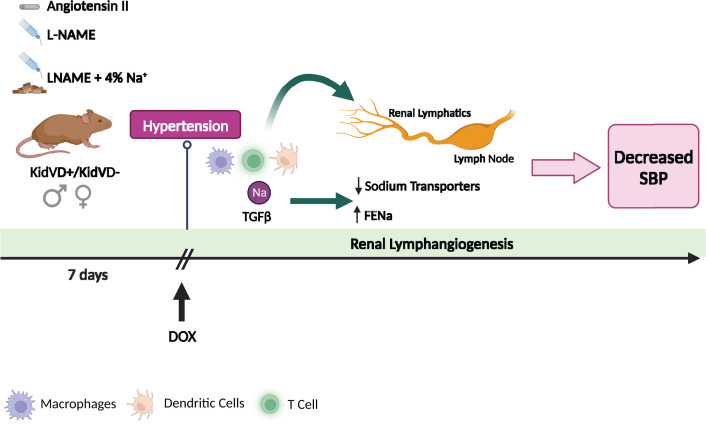
Representative scheme of the effects of renal lymphangiogenesis on models of hypertension Genetically induced renal lymphangiogenesis can decrease blood pressure in male and female mice. In the article published in *Clinical Science* by Goodlett and collaborators, hypertension was induced in male and female mice with inducible kidney-specific overexpression of VEGF-D (KidVD)+ and respective controls KidVD- via three different treatments: the chronic administration of Angiotensin II (subcutaneously), L-NAME (drinking water), or L-NAME (drinking water), followed by washout period and increased sodium in diet (chow). An increase in systolic blood pressure (hypertension) was seen after 7 days and this was associated with increased immune cell activation (macrophages, dendritic cells, and T cells) and the expression of inflammatory markers TGFβ. When renal lymphangiogenesis is induced via doxycycline treatment, a decrease in blood pressure was observed in association with an increase in the fractional excretion of sodium, decrease in the expression of sodium transporters as well as a decrease in renal-proinflammatory immune cell content. *Created with Biorender.com* using ‘*Mouse Experimental Timeline template*’ - modified.

The role of each subpopulation of immune cells to the beneficial effects of renal lymphangiogenesis on blood pressure levels is still unresolved. The authors speculate that the decrease in activated dendritic cells results from the decreased sodium content since dendritic cells can be activated by sodium [19]. Additionally, the decrease in myeloid cells was associated with increased expression of CCR7 and CCL21 in KidVD+ kidneys, suggesting increased traffic of immune cells to the lymph nodes as a mechanism to reduce blood pressure, and this implicates the activation of endothelial lymphatic cells as antigen presenters as part of the mechanisms whereby renal lymphangiogenesis reduces blood pressure.

The findings of Goodlett and collaborators are important as it supports the efficacy of renal lymphangiogenesis in reducing blood pressure in mice. The study also raised many questions on the molecular mechanisms whereby such process affects blood pressure levels as it involves sodium homeostasis and the mitigation of immune system activation. As improved sodium handling seems to be the foundational mechanism to decrease blood pressure, renal lymphangiogenesis may have implications in different models of hypertension that also affect sodium homeostasis. Furthermore, it may have implications in models of hypertension that affect renal function, such as endothelin 1-induced hypertension. Thus, renal lymphangiogenesis has the potential to have beneficial effects on blood pressure in models of hypertension that are not salt-sensitive. It would be important to identify the cell population responsible for the changes in blood pressure levels as well as to determine if the changes in cell populations observed by Goodlett and collaborators are also present in other models of hypertension. In addition, the investigation of the benefits of an advanced approach of targeting immune cells may be key to advance the treatment of resistant hypertension. Furthermore, whether renal lymphangiogenesis can decrease blood pressure in genetic animal models of hypertension is still unknown and should be explored as 95% of the cases in humans are idiopathic and not associated with the renal dysfunction seen in the studies models.

The potential of renal lymphangiogenesis as a treatment to human hypertension seems to be limited as the use of pro-lymphangiogenic agents would have a systemic effect. Although the interest in lymphatic tissue engineering and regeneration has gained attention in the past years, the options are limited and need clinical study validation. Potential available formulations to induce lymphangiogenesis could involve hydrogels, extracorporeal shockwave therapy and scaffolds [20]. The use of nanoparticle-directed lymphangiogenic factors that could be directed to the kidneys could increase the feasibility of targeted renal lymphangiogenesis as a treatment for resistant hypertension and this has started to be explored by the authors [21]. The work of Goodlett and collaborators has moved the field as it provided evidence of genetically induced renal lymphangiogenesis as an effective tool to reduce high blood pressure in mice and it sets a new start point for further studies to identify the molecular processes involved in this phenomenon.

## Data Availability

Not applicable.

## Open Access

Open access for the present article was enabled by the participation of University of Arizona in an all-inclusive *Read & Publish* agreement with Portland Press and the Biochemical Society under a transformative agreement with Individual.

## Abbreviations

A2HTN: Angiotensin II-induced hypertension
ENaC: epithelium sodium channel
eNOS: endothelial nitric oxide synthase
FENa: fractional excretion of sodium
KidVD+: kidney-specific overexpression of VEGF-D
MMF: mycophenolate mofetil
NCC: sodium-chloride symporter
Nhe3: sodium-hydrogen exchanger 3
LHTN: L-NAME iuduced hypertension
SSHTN: Salt-sensitive hypertension
